# Dietary cholesterol and egg intake are associated with the risk of gestational diabetes: a prospective study from Southwest China

**DOI:** 10.1186/s12884-022-04382-y

**Published:** 2022-01-17

**Authors:** Yiqi Zhang, Xi Lan, Fei Li, Hong Sun, Ju Zhang, Run Li, Yan Gao, Hongli Dong, Congjie Cai, Guo Zeng

**Affiliations:** 1 Department of Nutrition and Food Safety, West China School of Public Health and West China Fourth Hospital, Sichuan University, No. 16, Section 3, Renmin Nan Road, Chengdu, 610041 Sichuan China; 2 Department of Clinical Nutrition, Sichuan Provincial Hospital for Women and Children, Chengdu, 610000 Sichuan China; 3 Department of Obstetrics, Sichuan Provincial Hospital for Women and Children, Chengdu, 610000 Sichuan China

**Keywords:** Gestational diabetes mellitus, Dietary cholesterol, Eggs, Pregnancy

## Abstract

**Background:**

An increasing body of evidence suggests that cholesterol intake increases during pregnancy and may influence the risk of gestational diabetes mellitus (GDM). However, existing evidence remains controversial and limited. The present study aimed to determine the relation among dietary cholesterol, specifically egg consumption, in pregnant Chinese women and their risk of GDM.

**Methods:**

A population-based study that included 1617 pregnant women was conducted in 2017. At baseline, dietary information was collected by 24-hour dietary recalls over three days. GDM was diagnosed by a 75 g 2-hr oral glucose tolerance test (OGTT) at 24-28 weeks of gestation. Logistic regression models were used to examine the associations of dietary cholesterol and egg intake with GDM. In addition, path analysis including cholesterol intake, plasma lipid profiles and GDM risk was conducted.

**Results:**

The average total cholesterol intake was 340.8 mg/d, and cholesterol from eggs accounted for 59.2%. The odds ratio (OR) of GDM risk was 1.48 for the highest quartile of total cholesterol intake compared to the lowest quartile (95% CI 1.10-2.00; *P*_trend_ = 0.015) after adjustment for potential risk factors for GDM. Moreover, cholesterol from eggs rather than from other foods was positively associated with incident GDM (OR=1.09, 95% CI 1.03-1.17). Each additional egg consumed per day was positively correlated with a higher risk of GDM (OR=1.32, 95% CI 1.11-1.58). Path analysis indicated that cholesterol intake not only increased the risk of GDM by elevating plasma total cholesterol (TC), but also increased the risk of GDM through other non hyperlipidemia pathways.

**Conclusions:**

Maternal dietary cholesterol intake was significantly associated with incident GDM, and egg consumption was a major driver of the association in this population. More studies are needed to substantiate these findings and to explore the underlying mechanisms.

**Supplementary Information:**

The online version contains supplementary material available at 10.1186/s12884-022-04382-y.

## Background

Gestational diabetes mellitus (GDM), one of the most common obstetric complications, is a condition in which carbohydrate intolerance develops during pregnancy [1]. The prevalence of GDM is increasing worldwide, and GDM has been associated with an increased risk of maternal and neonatal adverse outcomes [2], as well as metabolic diseases such as obesity and type 2 diabetes mellitus (T2DM) in both mothers [3, 4] and their offspring [5, 6] in the long term. In 2015, the estimated cost of treating GDM and its complications in China was an astounding $5.59 billion [7].

Eggs and other cholesterol-rich foods are generally rich in high-quality protein and micronutrients; thus, pregnant women are encouraged to consume more. One survey in China showed that pregnant women's dietary cholesterol intake could reach 379 mg/d [8]. Therefore, the effects of cholesterol intake during pregnancy warrant further analysis. Previous studies have indicated that dietary cholesterol increases the risk of GDM [9, 10], and a meta-analysis of five longitudinal studies has shown that high cholesterol intake is positively associated with the risk of developing T2DM [11]. However, some studies have reported no association between cholesterol and T2DM or GDM [12–15]. Therefore, the relation between cholesterol intake and GDM is still unclear. In addition, extrapolating findings from previous studies to populations in other regions requires extra caution, given the wide diversity in dietary habits and disease incidence in different regions. To the best of our knowledge, no studies investigating the impact of dietary cholesterol on pregnant women have been reported in Southwest China.

Cholesterol, saturated fat, and animal protein often coexist in diets [10]. The interaction between dietary cholesterol and these nutrients with GDM remains uncertain. In addition, it is unknown whether plasma lipids play an important role in the association between dietary cholesterol and GDM, and it is also unclear whether different dietary cholesterol resources are important. Eggs are a major source of dietary cholesterol, and a medium-sized egg (edible part weight≈50 g) contains approximately 292 mg of cholesterol [16]. Several previous studies have suggested that egg consumption may be associated with GDM [8, 9]. However, previously published studies have yielded inconsistent findings [13–15]. The primary objective of the present study was to determine the association of dietary cholesterol and egg intake with GDM risk in pregnant Chinese women.

## Methods

### Study population and design

Participants for the present analysis were derived from a population-based prospective study conducted in Sichuan Provincial Hospital for Women and Children, Southwest China, which was designed to investigate the effects of maternal dietary, lifestyle and biochemical factors on the health of pregnant women. Pregnant women were invited to join this cohort during their first prenatal visit from February to September 2017. We recruited 1842 healthy women who met the following inclusion criteria: singleton pregnancy, gestational age ranging from 6 to 14 weeks, and absence of chronic metabolic diseases. When recruited, all participants completed an interviewer-administered questionnaire and were interviewed face-to-face by trained investigators about their lifestyle and dietary intake. If the pregnant woman reported severe vomiting, we did not collect her dietary information. This study was approved by the Institutional Ethics Committee of Sichuan University (reference k2017037) on March 14, 2017. All participants provided written informed consent.

In the present study, we considered the participants with the lowest quartile of cholesterol intake as the control group and the other participants as the exposure group. N1/P1 and N2/P2 are the numbers of participants/incidence rate of GDM in the exposure group and control group, respectively; we set N1/N2 = 3:1, aiming to explore the influence of three quartiles of cholesterol intake on GDM risk by comparing them to the lowest quartile of cholesterol intake (the control group). According to previous studies, the incidence of GDM in China was 17.5-18.9% [17, 18]. Compared to the lowest quintile, the ORs for GDM were 1.30-2.03 in other quintiles of cholesterol intake [8]. Therefore, we set P1=20%, and P2=12% in the present study. We then used PASS 15.0 (NCSS, LLC, Kaysville, UT, USA) to calculate the sample size (α = 0.05 (two-sided), 1-β = 0.90), and the minimum sample size (N = N1+N2) required was 1200 participants.

**Fig. 1:**
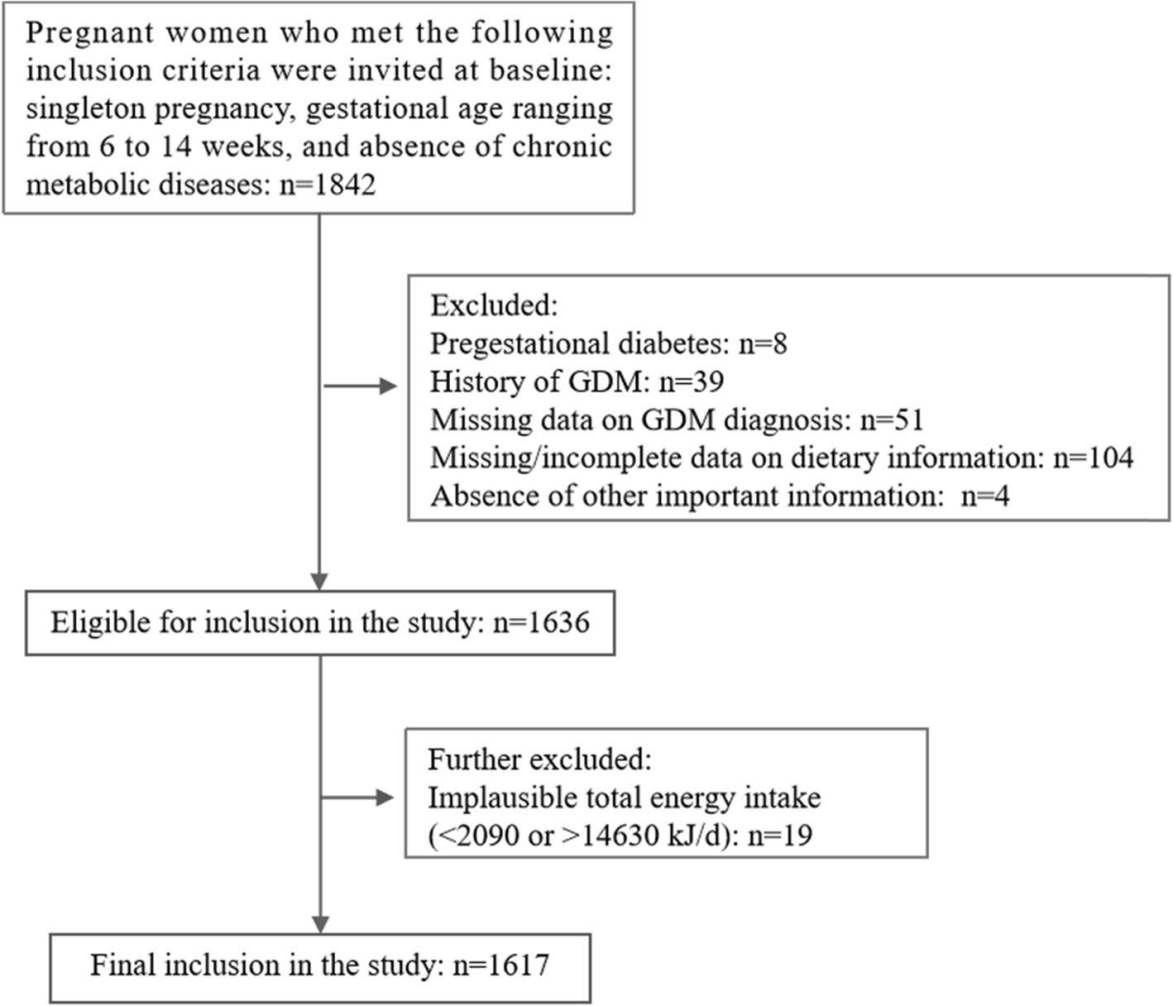
Flowchart for inclusion and exclusion of the study participants

Of these participants, 225 were excluded for pregestational diabetes (*n* = 8), history of GDM at baseline (*n* = 39), missing data on GDM diagnosis (*n* = 51), missing/incomplete data on dietary information (*n* = 104), and absence of other important information (*n* = 4). Pregnant women who reported implausible total energy intake (<2090 or >14630 kJ/d; *n* = 19) [19–21] were also excluded (*n* = 19). Therefore, 1617 pregnant women were eventually obtained for the statistical analyses. A flowchart providing a detailed breakdown of the sample size for this study is shown in Fig. 1.

### Dietary assessment

Dietary information was assessed by well-trained interviewers who had majored in nutrition through 24-h dietary recalls for three days, including two business days and one holiday. Detailed information about the types and amounts of food that participants ate and drank over the past 24-h period was obtained through face-to-face interviews at recruitment. Estimation tools such as standard serving bowls, cups, spoons, and food pictures of various portion sizes were used to assist quantification and reduce participant recollection bias. Dietary information for the remaining two days was collected from specialized interviewers by telephone in a structured manner.

Nutrition calculator v.2.7.3 was utilized to calculate the average daily intake of nutrients and energy based on the China Food Composition Database [16]. Dietary glycaemic load (GL) was calculated according to International Tables of Glycaemic Index [22] and the China Food Composition Database [16]. One egg was calculated as 50 g of edible part.

### Outcome assessment

Between gestational weeks 24 and 28, the participants were routinely screened using an oral glucose tolerance test (OGTT) to diagnose GDM. According to the criteria of the International Association of Diabetic Pregnancy Study Group guidelines [23], GDM was diagnosed if any of the glucose values on the 75g 2-hr OGTT met or exceeded the following thresholds: fasting plasma glucose ≥ 5.1 mmol/L (92 mg/dL), 1-h plasma glucose ≥ 10.0 mmol/L (180 mg/dL), or 2-h plasma glucose ≥ 8.5 mmol/L (153 mg/dL).

### Other variables

At baseline, information on maternal sociodemographic, clinical, and lifestyle factors was collected by trained investigators through face-to-face interviews with structured questionnaires. Prepregnancy weight was self-reported. Maternal height was measured by the standard method. The measurement accuracy was 0.1 cm, and the average of two consecutive measurements was used for analysis. Body mass index (BMI) was calculated by dividing height by the square of weight. Weight gain from pregnancy to OGTT (WGPO) was calculated as maternal weight at OGTT minus the prepregnancy weight. Physical activity was assessed using the pregnancy physical activity questionnaire (PPAQ), which has high reliability and validity [24]. A family history of diabetes was defined as having one or more first-degree relatives with diabetes. The plasma lipid profile concentration was measured at 12±1 gestational weeks after participants fasted overnight and within 2 hours after sampling. Fasting plasma glucose (FPG) was measured using the glucosidase method at 12±1 gestational weeks. Pregestational diabetes was diagnosed if the FPG ≥ 7.0 mmol/L (126 mg/dL )[25]. All tests were performed in the same laboratory and followed standard protocols.

### Statistical analyses

Continuous variables are presented as the means and standard deviations, and categorical variables are summarized as frequencies and percentages. Analysis of variance (ANOVA) and chi-square tests were used to compare the maternal characteristics according to cholesterol and egg consumption. Logistic regression models were used to examine the associations among cholesterol consumption, egg consumption, and GDM, and the results are presented as odds ratios (ORs) with 95% confidence intervals (CIs). We performed tests for linear trends by entering the median value of each category of cholesterol and egg intake as a continuous variable in the models. Generalized linear models were conducted to examine the association of total cholesterol and egg intake (per 100 mg/d and one egg/d increase, respectively) with FPG, 1-h postprandial plasma glucose (PPG), and 2-h PPG, and the results are presented as coefficients (*β*) with 95% CIs. We specifically selected confounding factors from the literature.

Nutrients correlated with dietary cholesterol (saturated fat, unsaturated fat, animal protein, and fibre) were adjusted individually or in combination with Model 2 covariates to evaluate the independent effect of cholesterol on GDM. To determine whether certain cholesterol-containing foods were major drivers of the association, we individually adjusted for eggs, red meat, fish/shellfish, poultry, total dairy products, and animal organs. To determine whether the effect of total cholesterol on GDM is related to elevated plasma lipids, the concentrations of plasma triglyceride (TG), total cholesterol (TC), low-density lipoprotein cholesterol (LDL-C), and high-density lipoprotein cholesterol (HDL-C) were further adjusted individually or in combination.

To evaluate the modification effect by potential risk factors for GDM, including maternal age (<30 or ≥30 years), prepregnancy BMI (<24 or ≥24 kg/m^2^), parity (primiparous or multiparous), physical activity (<Median or ≥Median), family history of diabetes (yes or no), concentration of plasma TG, TC, LDL-C, and HDL-C (<Median or ≥Median), we conducted stratified analyses by these potential factors and estimated *P* values for interaction terms. In addition, path analysis, including total cholesterol intake, plasma TG, TC, LDL-C, HDL-C levels, and GDM risk, was conducted. Age and prepregnancy BMI were included as possible confounding factors. All analyses were performed using the statistical package R (http://www.R-project.org; 3.4.3 version) and Empower (R) (www.empowerstats.com, X&Y Solutions, Inc. Boston MA). Statistical significance was accepted at a two-sided *P* value of <0.05.

## Results

### Study population characteristics

The basic characteristics and dietary intake of the 1617 pregnant women are shown in Table 1 and Table S1. Most of the pregnant women were Han Chinese (97.4%). The average total cholesterol intake was 340.8 mg/day. Cholesterol from eggs accounted for 59.2% followed by red meat (12.1%), poultry (6.4%), fish/shellfish (6.4%), dairy products (6.2%), animal organs (2.8%), and other foods (6.9%).

**Table 1:** Characteristics and dietary intake of participants according to quartile of total cholesterol intake

Characteristic	Overall		Quartile of total cholesterol intake (mg/d)								*P* -value
			Q1(≤186.0)		Q2(186.1-332.0)		Q3(332.1-464.0)		Q4(>464.0)		
**N**	1617		408		404		401		404		
Age, years (mean, SD)	28.6	4.1	28.0	3.8	28.5	3.9	29.0	4.4	28.9	4.1	0.001
Prepregnancy BMI, kg/m^2^ (mean, SD)	21.1	3.1	21.2	3.2	21.1	3.1	21.3	3.0	20.9	3.0	0.249
Parity (N, %)											
Primiparous	1157	71.6	290	71.1	286	70.8	293	73.1	288	71.3	0.890
Multiparous	460	28.4	118	28.9	118	29.2	108	26.9	116	28.7	
Family history of diabetes (N, %)	162	10.0	40	9.8	40	9.9	41	10.2	41	10.1	0.997
Education (N, %)											
High school or lower	387	23.9	103	25.2	109	27.0	96	23.9	79	19.6	<0.001
Junior college	590	36.5	175	42.9	135	33.4	146	36.4	134	33.2	
University or higher	640	39.6	130	31.9	160	39.6	159	39.7	191	47.3	
Monthly income of the family per capita, yuan (N, %)											
≤5000	535	33.1	147	36.0	125	30.9	134	33.4	129	31.9	0.215
5000~9999	743	45.9	192	47.1	191	47.3	184	45.9	176	43.6	
≥10000	339	21.0	69	16.9	88	21.8	83	20.7	99	24.5	
Smoking before pregnancy (N, %)	61	3.8	17	4.2	10	2.5	12	3.0	22	5.5	0.119
Alcohol consumption before pregnancy (N, %)	131	8.1	34	8.3	23	5.7	28	7.0	46	11.4	0.021
WGPO, kg (mean, SD)	6.20	3.31	5.64	3.26	6.43	3.25	6.27	3.26	6.44	3.40	0.001
Physical activity, MET^-h^ wk^-1^ (mean, SD)	106.8	46.4	105.3	48.8	108.7	49.0	105.8	41.9	107.4	45.5	0.710
Plasma TG, mmol/L (mean, SD)	1.32	0.50	1.30	0.47	1.31	0.45	1.36	0.52	1.30	0.57	0.257
Plasma TC, mmol/L (mean, SD)	4.14	0.64	4.05	0.66	4.13	0.61	4.17	0.63	4.21	0.67	0.002
Plasma LDL-C, mmol/L (mean, SD)	2.05	0.9	2.03	0.52	2.05	0.48	2.06	0.48	2.04	0.48	0.932
Plasma HDL-C, mmol/L (mean, SD)	1.67	0.34	1.60	0.34	1.66	0.34	1.68	0.32	1.75	0.35	<0.001
GDM (N, %)	571	35.3	118	28.9	146	36.1	151	37.7	156	38.6	0.016
**Dietary intake**											
Eggs, number/d (mean, SD)	0.71	0.58	0.12	0.21	0.49	0.29	0.89	0.31	1.34	0.56	<0.001
Red meats, g/d (mean, SD)	59.5	50.5	39.3	39.0	59.5	41.7	61.0	51.1	78.3	59.7	<0.001
Poultry, g/d (mean, SD)	12.5	29.2	6.3	17.7	11.2	22.4	13.5	29.2	19.3	40.8	<0.001
Fish/shellfish, g/d (mean, SD)	18.6	41.8	6.9	19.3	14.1	33.5	17.6	41.6	35.8	57.8	<0.001
Total dairy products, g/d (mean, SD)	138.5	132.8	93.8	116.2	132.6	129.5	149.4	124.7	178.7	145.0	<0.001
Animal organs, g/d (mean, SD)	5.4	17.3	3.2	14.8	4.6	11.5	4.5	13.6	9.1	25.4	<0.001
Dietary glycaemic load, g/d (mean, SD)	153.3	59.4	147.3	58.7	152.7	53.6	158.4	67.6	154.8	56.7	0.061
Energy intake, kcal/d (mean, SD)	1848.1	530.5	1600.1	486.6	1819.1	510.2	1901.2	475.4	2074.9	535.9	<0.001
Saturated fat, unsaturated fat, g/d (mean, SD)	68.8	24.8	53.0	19.1	66.6	23.5	72.0	20.3	83.6	25.6	<0.001
Animal protein, g/d (mean, SD)	25.4	15.4	12.8	8.2	22.7	10.4	27.3	12.2	39.1	16.4	<0.001
Fibre, g/d (mean, SD)	12.9	6.3	11.9	6.5	12.9	6.7	13.0	5.8	13.7	5.9	<0.001

*BMI* body mass index, *WGPO* Weight gain from pregnancy to OGTT, *TG* triglyceride, *TC* total cholesterol, *HDL-C* high-density lipoprotein cholesterol, *LDL-C* low-density lipoprotein cholesterol, *GDM* gestational diabetes mellitus, *MET* metabolic equivalent

### Associations between maternal cholesterol intake and GDM

After adjusting for potential confounders, each additional 100 mg/d of total dietary cholesterol was significantly and positively associated with a higher risk of GDM (OR=1.07, 95% CI 1.02-1.13; Table 2). The ORs for GDM were 1.39 (95% CI: 1.03, 1.88), 1.40 (95% CI: 1.04, 1.89) and 1.48 (95% CI: 1.10, 2.00) for successive quartiles of total cholesterol intake (*P*_trend_=0.015) compared to the lowest quartile (Table 2). These associations remained significant after further adjustment for plasma lipid profiles, dietary fats, animal protein, fibre, red meats, poultry, fish/shellfish, total dairy products, and animal organs. However, the association between total cholesterol consumption and the risk of GDM was no longer significant after adjustment for egg consumption (OR=1.01, 95% CI 0.92-1.11; Table 2). The ORs were 1.26 (95% CI: 0.91, 1.73), 1.13 (95% CI: 0.77, 1.64), and 1.05 (95% CI: 0.66, 1.66) for successive quartiles of total cholesterol intake after adjustment for egg consumption (*P*_trend_=0.941). The associations between total cholesterol intake and blood glucose levels are shown in Table S2.

**Table 2:** Associations between total cholesterol intake per day and GDM

	Continuous variable (each additional 100 mg/d)		Quartile of total cholesterol intake (mg/d)								*P* for trend
			Q1		Q2		Q3		Q4		
	OR	95%CI	OR	95%CI	OR	95%CI	OR	95%CI	OR	95%CI	
Median, mg/d	332.0		103.0		268.0		394.0		571.5		-
Model 1^a^	1.08	1.03-1.14	1.00	-	1.39	1.04-1.87	1.48	1.11-1.99	1.55	1.15-2.07	0.004
Model 2^b^	1.07	1.02-1.13	1.00	-	1.39	1.03-1.88	1.40	1.04-1.89	1.48	1.10-2.00	0.015
Model 2 plus nutrients ^c^											
Saturated fat, unsaturated fat	1.07	1.01-1.13	1.00	-	1.37	1.01-1.86	1.37	1.00-1.87	1.42	1.02-1.98	0.049
Animal protein	1.10	1.03-1.18	1.00	-	1.43	1.05-1.96	1.45	1.04-2.00	1.61	1.11-2.34	0.019
Fibre	1.07	1.02-1.13	1.00	-	1.40	1.03-1.89	1.40	1.04-1.90	1.49	1.10-2.01	0.014
All previous nutrients	1.10	1.02-1.17	1.00	-	1.42	1.04-1.94	1.42	1.02-1.97	1.59	1.09-2.32	0.024
Model 2 plus cholesterol-containing foods ^d^											
Eggs	1.01	0.92-1.11	1.00	-	1.26	0.91-1.73	1.13	0.77-1.64	1.05	0.66-1.66	0.941
Red meats	1.08	1.02-1.14	1.00	-	1.39	1.02-1.88	1.41	1.04-1.91	1.51	1.11-2.07	0.013
Poultry	1.08	1.02-1.14	1.00	-	1.38	1.02-1.87	1.41	1.04-1.91	1.52	1.12-2.06	0.010
Fish/shellfish	1.07	1.02-1.13	1.00	-	1.39	1.03-1.89	1.40	1.04-1.90	1.48	1.09-2.02	0.018
Total dairy products	1.07	1.01-1.12	1.00	-	1.38	1.02-1.86	1.37	1.01-1.86	1.44	1.06-1.96	0.031
Animal organs	1.07	1.02-1.13	1.00	-	1.37	1.01-1.85	1.38	1.02-1.87	1.46	1.08-1.98	0.019
Model 2 plus plasma lipid profiles (mmol/L) ^e^											
TG	1.08	1.02-1.14	1.00	-	1.41	1.03-1.92	1.37	1.00-1.87	1.54	1.10-2.14	0.021
TC	1.08	1.02-1.14	1.00	-	1.40	1.03-1.91	1.38	1.01-1.88	1.49	1.07-2.08	0.029
LDL-C	1.08	1.02-1.15	1.00	-	1.41	1.04-1.92	1.39	1.02-1.91	1.53	1.10-2.13	0.019
HDL-C	1.08	1.02-1.14	1.00	-	1.40	1.03-1.91	1.38	1.01-1.89	1.50	1.08-2.09	0.027
All previous measurements	1.08	1.01-1.14	1.00	-	1.40	1.03-1.91	1.36	1.00-1.86	1.51	1.08-2.11	0.028

*TG* triglyceride, *TC* total cholesterol, *HDL-C* high-density lipoprotein cholesterol, *LDL-C* low-density lipoprotein cholesterol, *GDM* gestational diabetes mellitus

^a^ Crude model

^b^ Adjusted for energy intake, age, prepregnancy BMI, education, income of family, parity, family history of diabetes, smoking before pregnancy, alcohol consumption before pregnancy, physical activity, dietary glycaemic load, weight gain from pregnancy to OGTT

^c^ Nutrients correlated with dietary cholesterol (saturated fat, unsaturated fat, trans fat, animal protein and fibre) were adjusted individually or in combination, in addition to Model 2 covariates

^d^ To determine if certain cholesterol-containing foods were major determinants for the associations, eggs, red meat, poultry, fish and shellfish, total dairy products and edible offal were adjusted individually in addition to Model 2 covariates

^e^ To determine whether the effect of total cholesterol on GDM is related to the increase of plasma lipid profiles, the concentrations of plasma TG, TC, LDL-C and HDL-C were further adjusted individually or in combination in addition to Model 2 covariates

We further divided total cholesterol into cholesterol from eggs and cholesterol from other foods (except eggs) to explore the potentially unique contributions of egg intake. Cholesterol from eggs was positively associated with incident GDM in fully adjusted models. The ORs for GDM were 1.14 (95% CI: 0.90, 1.46) and 1.43 (95% CI: 1.08, 1.90) for successive tertiles of cholesterol from egg intake (*P*_trend_=0.019) compared to the lowest tertiles (Table 3). However, the association between cholesterol from other foods (except eggs) and GDM was not significant (*P* > 0.05; Table 3).

**Table 3:** Associations of cholesterol from eggs and other foods per day with GDM

	Continuous variable (each additional 100 mg/d)		Tertiles of cholesterol intake (mg/d)						*P* for trend
			Q1		Q2		Q3		
	OR	95%CI	OR	95%CI	OR	95%CI	OR	95%CI	
**Cholesterol from eggs**									
Median, mg/d	194.7		107.0		127.3		128.3		
Model 1^a^	1.11	1.04-1.17	1.00	-	1.22	0.97-1.55	1.50	1.15-1.97	0.003
Model 2^b^	1.09	1.03-1.17	1.00	-	1.14	0.90-1.46	1.43	1.08-1.90	0.019
Model 2 plus nutrients ^c^									
Saturated fat, unsaturated fat	1.09	1.03-1.16	1.00	-	1.12	0.88-1.44	1.40	1.05-1.86	0.027
Animal protein	1.10	1.03-1.17	1.00	-	1.14	0.89-1.47	1.42	1.07-1.90	0.022
Fibre	1.09	1.03-1.16	1.00	-	1.14	0.89-1.45	1.42	1.07-1.88	0.021
All previous nutrients	1.10	1.03-1.17	1.00	-	1.13	0.88-1.45	1.42	1.06-1.90	0.025
**Cholesterol from other foods** ^**d**^									
Median, mg/d	121.0		51.0		121.0		216.0		
Model 1^a^	1.02	0.93-1.13	1.00	-	1.34	1.04-1.73	1.30	1.00-1.59	0.047
Model 2^b^	0.99	0.88-1.12	1.00	-	1.33	0.98-1.80	1.28	0.93-1.45	0.102
Model 2 plus nutrients ^c^									
Saturated fat, unsaturated fat	0.96	0.85-1.09	1.00	-	1.26	0.92-1.72	1.23	0.88-1.40	0.240
Animal protein	0.95	0.81-1.12	1.00	-	1.40	0.97-2.03	1.36	0.96-1.58	0.103
Fibre	0.98	0.87-1.11	1.00	-	1.32	0.97-1.02	1.28	0.92-1.44	0.126
All previous nutrients	0.93	0.79-1.10	1.00	-	1.34	0.92-1.94	1.31	0.90-1.54	0.181

*GDM* gestational diabetes mellitus

^a^ Crude model

^b^ Adjusted for energy intake, age, prepregnancy BMI, education, income of family, parity, family history of diabetes, smoking before pregnancy, alcohol consumption before pregnancy, physical activity, dietary glycaemic load, weight gain from pregnancy to OGTT

^c^ Nutrients correlated with dietary cholesterol (saturated fat, unsaturated fat, trans fat, animal protein, and fibre) were adjusted individually or in combination in addition to Model 2 covariates.

^d^ Other foods included red meat, poultry, fish and shellfish, dairy products, animal organs and the others (except eggs)

### Associations between maternal egg intake and GDM

After adjusting for potential confounders, each additional egg consumed per day was significantly and positively associated with a higher risk of incident GDM (OR=1.32, 95% CI 1.11-1.58; Table 4). The ORs for GDM were 1.00 (95% CI reference), 1.48 (95% CI 1.11, 1.99), 1.39 (95% CI 1.03, 1.87), and 1.59 (95% CI 1.18, 2.14) for successive quartiles of egg intake (*P*_trend_=0.006) (Table 4). These associations remained significant after further adjustment for plasma lipid profiles, dietary fats, animal protein, and fibre.

**Table 4:** Associations between maternal dietary egg intake per day and GDM

	Continuous variable (each additional egg/d)		Quartile egg intake (number/d)								*P* for trend
			Q1		Q2		Q3		Q4		
	OR	95%CI	OR	95%CI	OR	95%CI	OR	95%CI	OR	95%CI	
Median, number/d	0.7		0		0.5		1.0		1.3		-
Model 1^a^	1.36	1.14-1.62	1.00	-	1.48	1.11-1.98	1.46	1.09-1.96	1.64	1.22-2.20	0.002
Model 2^b^	1.32	1.11-1.58	1.00	-	1.48	1.11-1.99	1.39	1.03-1.87	1.59	1.18-2.14	0.006
Model 2 plus nutrients^c^											
Saturated fat, unsaturated fat	1.30	1.08-1.56	1.00	-	1.47	1.09-1.97	1.35	1.00-1.84	1.54	1.13-2.09	0.013
Animal protein	1.33	1.10-1.60	1.00	-	1.48	1.11-1.99	1.39	1.02-1.89	1.59	1.16-2.17	0.008
Fibre	1.32	1.11-1.58	1.00	-	1.48	1.11-1.99	1.39	1.03-1.88	1.59	1.18-2.15	0.005
All previous nutrients	1.32	1.10-1.60	1.00	-	1.48	1.10-1.98	1.38	1.02-1.88	1.58	1.16-2.17	0.009
Model 2 plus plasma lipid profiles (mmol/L) ^d^											
TG	1.30	1.10-1.60	1.00	-	1.57	1.16-2.12	1.46	1.07-1.99	1.65	1.20-2.25	0.009
TC	1.31	1.09-1.58	1.00	-	1.61	1.19-2.18	1.45	1.06-1.98	1.64	1.20-2.24	0.011
LDL-C	1.33	1.11-1.60	1.00	-	1.62	1.20-2.19	1.46	1.07-1.99	1.68	1.23-2.30	0.007
HDL-C	1.31	1.09-1.58	1.00	-	1.60	1.19-2.16	1.44	1.06-1.96	1.64	1.20-2.25	0.011
All previous measurements	1.30	1.08-1.56	1.00	-	1.58	1.17-2.13	1.46	1.07-1.99	1.63	1.19-2.23	0.011

*TG* triglyceride, *TC* total cholesterol, *HDL-C* high-density lipoprotein cholesterol, *LDL-C* low-density lipoprotein cholesterol, *GDM* gestational diabetes mellitus

^a^ Crude model

^b^ Adjusted for energy intake, age, prepregnancy BMI, education, income of family, parity, family history of diabetes, smoking before pregnancy, alcohol consumption before pregnancy, physical activity, dietary glycaemic load, weight gain from pregnancy to OGTT

^c^ Nutrients correlated with egg (saturated fat, unsaturated fat, trans fat, animal protein and fibre) were adjusted individually or in combination, in addition to Model 2 covariates.

^d^ To determine whether the effect of egg intake on GDM is related to the increase of plasma lipid profiles, the concentrations of plasma TG, TC, LDL-C and HDL-C were further adjusted individually or in combination

### Subgroup analyses

The association between total cholesterol intake (per 100 mg/day) and incident GDM was stronger in participants with plasma TG, TC, and LDL-C levels above the median (*P* interaction=0.019, 0.006 and 0.022, respectively; Table 5) as well as stronger in participants over the age of 30 (*P* interaction=0.048; Table 5). The association of both total cholesterol and egg intake with GDM risk was not significantly modified by prepregnancy BMI, parity, physical activity, family history of diabetes, or plasma HDL-C level (*P* interaction >0.05) (Table 5).

**Table 5:** Stratified analyses of potential modification effect for the impact of total cholesterol and egg intake on GDM

Stratification factors		Total cholesterol intake (each additional 100 mg/d) ^a^			Egg intake (each additional egg/d) ^a^		
	N	OR	95%CI	*P* for interaction	OR	95%CI	*P* for interaction
All participants	1617	1.07	1.02-1.13	-	1.32	1.11-1.58	-
Age (years)							
<30	969	1.02	0.95-1.10	0.048	1.12	0.89-1.42	0.036
≥30	648	1.24	1.12-1.32		1.66	1.24-2.22	
Prepregnancy BMI (kg/m^2^)							
<24	1369	1.07	1.02-1.14	0.985	1.31	1.08-1.58	0.676
≥24	248	1.06	0.91-1.24		1.50	0.86-2.61	
Parity							
Primiparous	1157	1.06	1.00-1.13	0.575	1.28	1.03, 1.58	0.673
Multiparous	460	1.11	1.0-1.22		1.46	1.04, 2.05	
Physical activity (MET^-h^ wk^-1)^							
<Median	809	1.48	1.14-1.93	0.396	1.11	1.02, 1.20	0.262
≥Median	808	1.21	0.94-1.54		1.05	0.98, 1.13	
Family history of diabetes							
Yes	162	1.03	0.87-1.21	0.994	1.16	0.67, 2.00	0.964
No	1455	1.07	1.02-1.13		1.32	1.09, 1.60	
Plasma TG (mmol/L)							
<Median	828	1.06	0.97-1.15	0.019	1.29	0.97-1.71	0.260
≥Median	789	1.10	1.01-1.19		1.33	1.04-1.70	
Plasma TC (mmol/L)							
<Median	894	1.03	0.95-1.11	0.006	1.19	0.93-1.53	0.011
≥Median	723	1.14	1.04-1.25		1.51	1.14-2.01	
Plasma LDL-C (mmol/L)							
<Median	893	1.02	0.95-1.09	0.022	1.10	0.86-1.40	0.023
≥Median	724	1.15	1.07-1.24		1.68	1.28-2.21	
Plasma HDL-C (mmol/L)							
<Median	808	1.02	0.94-1.10	0.119	1.17	0.90-1.52	0.241
≥Median	809	1.11	1.04-1.20		1.47	1.14-1.89	

*BMI* body mass index, *TG* triglyceride, *TC* total cholesterol, *HDL-C* high-density lipoprotein cholesterol, *LDL-C* low-density lipoprotein cholesterol, *GDM* gestational diabetes mellitus, *MET* metabolic equivalent

^a^ Adjusted for energy intake, age, prepregnancy BMI, education, income of family, parity, family history of diabetes, smoking before pregnancy, alcohol consumption before pregnancy, physical activity, dietary glycemic load, weight gain from pregnancy to OGTT

### Path analysis

**Fig. 2:**
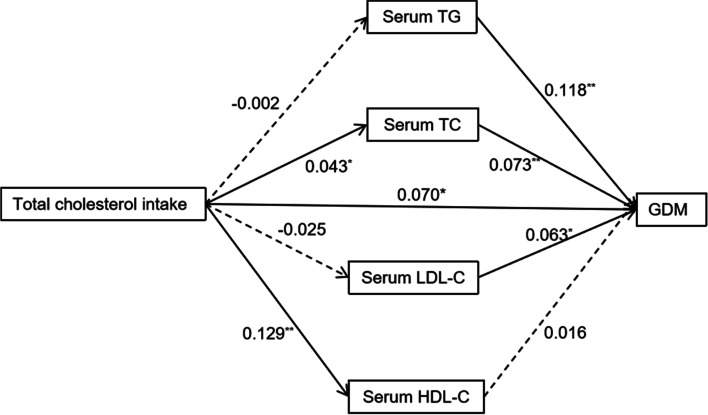
Results of path analysis with cholesterol intake (mg/d), lipid profiles (mmol/L) and GDM risk adjusting for maternal age and prepregnancy BMI. The solid arrows represent statistically significant direct paths, and the dotted arrows represent nonsignificant direct paths. The numbers beside the arrows show the standardized path coefficients. ^*^p < 0.05, ^**^p < 0.01. TG, triglyceride; TC, total cholesterol; HDL-C, high-density lipoprotein cholesterol; LDL-C, low-density lipoprotein cholesterol; GDM, gestational diabetes mellitus; BMI, body mass index

Figure 2 shows the results of path analysis, including total cholesterol intake, plasma TG, TC, LDL-C, HDL-C levels, and GDM risk. The paths from total cholesterol intake to the concentrations of TC and HDL-C as well as the paths from the concentrations of TG, TC, and LDL-C neuroticism to GDM risk were statistically significant (*P*<0.05). In addition, the direct path from total cholesterol intake to GDM risk was also statistically significant (standardized path coefficient: 0.070, *P* = 0.010).

## Discussion

In the present study, we observed a significant positive association between total cholesterol intake during pregnancy and incident GDM after adjusting for potential risk factors for GDM, including fat amount and animal protein. Similarly, cholesterol intake before pregnancy positively correlated with the risk of GDM in a large cohort study, including 13475 women of childbearing age in the United States [26]. Moreover, Qiu et al. [9] found an increased GDM risk of 1.35 times in mothers with cholesterol intake of more than 294 mg/d compared to mothers with dietary cholesterol intake of less than 151 mg/d. Wu et al. [8] reported similar results in a cohort study involving 2124 pregnant Chinese women. However, no correlation between cholesterol intake and GDM was found in three case-control studies in Iran [13–15], which may account for the low level of cholesterol intake.

It is widely acknowledged that the pathogenesis of GDM is similar to that of T2DM. Previous studies have shown that cholesterol intake is positively associated with T2DM [11, 27]. However, neither dietary cholesterol nor egg intake was associated with T2DM in a large cohort study of 3898 people over 65 years of age with an average follow-up of 11.3 years in the United States [12]. At present, much controversy surrounds the correlation between dietary cholesterol and T2DM.

No association between dietary cholesterol and GDM was found after adjustment for egg intake rather than other foods, suggesting that eggs maybe the major driver of the association. We further explored the correlation of cholesterol from eggs and other foods (except eggs) with GDM. Cholesterol from eggs was positively associated with incident GDM, while cholesterol from the other foods was not associated with incident GDM. We speculated that the inconsistent results may be due to differences in the proportion of dietary cholesterol sources. In the present study, cholesterol from eggs accounted for 59.2% of dietary cholesterol. Moreover, we further explored the correlation between egg intake and GDM and found that each additional egg consumed per day was significantly and positively associated with GDM after adjusting for potential risk factors for GDM, including fat amount and animal protein. As a high cholesterol food, limiting egg intake to a reasonable level in pregnant women may help reduce the risk of GDM.

The mechanisms underlying the effect of dietary cholesterol on blood glucose metabolism have not been fully determined. Many studies have documented that plasma lipids play an important role in blood glucose homeostasis [28–30]. Some animal experiments have found that elevated plasma cholesterol levels may activate the proinflammatory signal cascade, and excessive cholesterol intake may elevate the serum levels of the amyloid A inflammatory marker, suggesting that excessive cholesterol intake increases serum cholesterol concentration, promotes the inflammatory response, and leads to insulin resistance [31, 32]. Another study has suggested that cholesterol accumulation in islets may cause damage to the function of pancreatic β-cells [33]. In the present study, after adjustment for plasma lipid levels, the association was still present, suggesting that the effect of dietary cholesterol on blood glucose is not entirely due to hyperlipidaemia. Path analysis showed that cholesterol intake not only increased the risk of GDM by elevating plasma TC, but also increased the risk of GDM through other pathways.

The 2015-2020 Dietary Guidelines for Americans [34] does not specify a daily cholesterol intake limit but recommends consuming as little dietary cholesterol as possible while consuming a healthy eating pattern. In 2013, the Chinese Nutrition Society removed the upper limit of recommended dietary cholesterol intake from the DRIs [35]. These recommendations might lead to excessive dietary cholesterol and egg consumption in the general population, leading to an increase in the incidence of GDM or T2DM. The dietary cholesterol intake of Chinese adults has gradually increased over the past 20 years from 165.8 mg/d in 1991 to 266.3 mg/d in 2011 [36]. In the present study, the average dietary cholesterol intake in early pregnancy, mainly from egg consumption, was 340.8 mg/d. Current evidence suggests that overconsumption of cholesterol can have deleterious effects in pregnant women. Thus, cholesterol intake should be limited to reasonable levels in pregnant women to reduce the risks of GDM.

There were several strengths in the present study. Our prospective study provided evidence of the association between dietary cholesterol intake and GDM, and it avoided reverse causality bias. Because cholesterol-containing foods are usually rich in saturated fat and animal protein, adjustment for these factors substantiated the effect of cholesterol on GDM. In addition, evidence from our study suggested that dietary cholesterol leads to elevated blood glucose by not only affecting lipid metabolism but other pathways as well. Although this finding needs further validation, our study provides a basis for future studies exploring the mechanisms underlying the effect of dietary cholesterol on blood glucose metabolism.

The present study had several limitations that should be considered. First, the participants in this study only came from a hospital with good medical resources in Southwest China, which may lead to an unrepresentative sample. Accordingly, similar studies need to be conducted in other populations. In addition, consistent with other observational studies, the possibility of residual confounding from unmeasured or unknown covariates cannot be ruled out, although adjustment for multiple potential confounders was included in the analyses. Moreover, the three-day 24-h dietary recalls did not assess the frequency of food intake and, hence, did not reflect a long-term diet. However, estimates of the absolute magnitude of energy and food intake recalls were generally choice methods. If data documenting at least two days of food intake per person were available, the true distribution of food intake for the population could be estimated [37].

## Conclusions

During pregnancy, high dietary cholesterol consumption was significantly associated with a higher risk of GDM, and egg consumption was a major driver of the association in this population. More studies are needed to validate these findings and to explore the underlying mechanisms. The present study indicated that overconsumption of cholesterol warrants concern among pregnant women. Maternal cholesterol intake should be limited to reasonable levels to minimize the risks of GDM.

## Supplementary Information

**Additional file 1: Table S1.** Characteristics and dietary intake of participants according to quartile of egg intake. **Table S2.** Associations of total cholesterol and egg intake with blood glucose levels

## Data Availability

The datasets used and/or analysed during the current study are available from the corresponding author on reasonable request.

## Abbreviations

GDM: Gestational diabetes mellitus
T2DM: Type 2 diabetes mellitus
OGTT: Oral glucose tolerance test
CVD: Cardiovascular disease
DRIs: Dietary Reference Intakes
GL: Glycemic load
BMI: Body Mass Index
WGPO: Weight gain from pregnancy to OGTT
PPAQ: Pregnancy physical activity questionnaire
ANOVA: Analysis of variance
OR: Odds ratio
CI: Confidence intervals
FPG: Fasting plasma glucose
PPG: Postprandial plasma glucose
TG: Triglyceride
TC: Total cholesterol
LDL-C: Low-density lipoprotein cholesterol
HDL-C: High-density lipoprotein cholesterol

## References

[1] American Diabetes A (2014). Diagnosis and classification of diabetes mellitus. Diabetes Care.

[2] Farrar D, Simmonds M, Bryant M, Sheldon TA, Tuffnell D, Golder S (2016). Hyperglycaemia and risk of adverse perinatal outcomes: systematic review and meta-analysis. BMJ..

[3] Li Z, Cheng Y, Wang D, Chen H, Chen H, Ming WK (2020). Incidence rate of type 2 diabetes mellitus after gestational diabetes mellitus: a systematic review and meta-analysis of 170,139 women. J Diabetes Res.

[4] Song C, Lyu Y, Li C, Liu P, Li J, Ma RC (2018). Long-term risk of diabetes in women at varying durations after gestational diabetes: a systematic review and meta-analysis with more than 2 million women. Obes Rev.

[5] Nijs H, Benhalima K (2020). Gestational diabetes mellitus and the long-term risk for glucose intolerance and overweight in the offspring: a narrative review. J Clin Med.

[6] Bianco ME, Josefson JL (2019). Hyperglycemia during pregnancy and long-term offspring outcomes. Curr Diab Rep.

[7] Xu T, Dainelli L, Yu K, Ma L, Silva Zolezzi I, Detzel P (2017). The short-term health and economic burden of gestational diabetes mellitus in China: a modelling study. BMJ Open.

[8] Wu Y, Sun G, Zhou X, Zhong C, Chen R, Xiong T (2020). Pregnancy dietary cholesterol intake, major dietary cholesterol sources, and the risk of gestational diabetes mellitus: a prospective cohort study. Clin Nutr.

[9] Qiu C, Frederick IO, Zhang C, Sorensen TK, Enquobahrie DA, Williams MA (2011). Risk of gestational diabetes mellitus in relation to maternal egg and cholesterol intake. Am J Epidemiol.

[10] Gao F, Cui CY. Dietary cholesterol intake and risk of gestational diabetes mellitus: a meta-analysis of observational studies. J Am Coll Nutr. 2021. 10.1080/07315724.2020.1844605. 10.1080/07315724.2020.1844605 33416437

[11] Tajima R, Kodama S, Hirata M, Horikawa C, Fujihara K, Yachi Y (2014). High cholesterol intake is associated with elevated risk of type 2 diabetes mellitus - a meta-analysis. Clin Nutr.

[12] Djousse L, Kamineni A, Nelson TL, Carnethon M, Mozaffarian D, Siscovick D (2010). Egg consumption and risk of type 2 diabetes in older adults. Am J Clin Nutr.

[13] Parsanahad M, Karandish M, Shahbazian N, al. e. (2013). The relationship between egg consumption during pregnancy and risk of gestational diabetes mellitus. J Health Res.

[14] Milajerdi A, Tehrani H, Haghighatdoost F, Larijani B, Surkan P, Azadbakht L (2018). Associations between higher egg consumption during pregnancy with lowered risks of high blood pressure and gestational diabetes mellitus. Int J Vitam Nutr Res.

[15] Karandish M, Parsanahad M, Shahbazian N, Haghighizadeh M (2013). Relationship between egg consumption and gestational diabetes mellitus. J Gastroenterol Hepatol.

[16] Yang Y, Wang G, Pan X (2009). China food composition.

[17] Zhu WW, Fan L, Yang HX, Kong LY, Su SP, Wang ZL (2013). Fasting plasma glucose at 24-28 weeks to screen for gestational diabetes mellitus: new evidence from China. Diabetes Care.

[18] Wei Y, Yang H, Zhu W, Yang H, Li H, Jie Y (2014). International Association of Diabetes and Pregnancy Study Group criteria is suitable for gestational diabetes mellitus diagnosis:further evidence from China. Chin Med J.

[19] Rhee JJ, Sampson L, Cho E, Hughes MD, Hu FB, Willett WC (2015). Comparison of methods to account for implausible reporting of energy intake in epidemiologic studies. Am J Epidemiol.

[20] Mendez MA, Popkin BM, Buckland G, Schroder H, Amiano P, Barricarte A (2011). Alternative methods of accounting for underreporting and overreporting when measuring dietary intake-obesity relations. Am J Epidemiol.

[21] Huang TT, Roberts SB, Howarth NC, McCrory MA (2005). Effect of screening out implausible energy intake reports on relationships between diet and BMI. Obes Res.

[22] Atkinson FS, Foster-Powell K, Brand-Miller JC (2008). International tables of glycemic index and glycemic load values: 2008. Diabetes Care.

[23] Metzger BE, Gabbe SG, Persson B, Buchanan TA, Catalano PA, Damm P (2010). International association of diabetes and pregnancy study groups recommendations on the diagnosis and classification of hyperglycemia in pregnancy. Diabetes Care.

[24] Chasan-Taber L, Schmidt MD, Roberts DE, Hosmer D, Markenson G, Freedson PS (2004). Development and validation of a pregnancy physical activity questionnaire. Med Sci Sports Exerc.

[25] Department of Obstetrics and Gynecology CMA, Perinatal Medicine Branch of Chinese Medical Association (2014). Guidelines for diagnosis and treatment of pregnancy complicated with diabetes. Chinese J Obstetr Gynecol.

[26] Bowers K, Tobias DK, Yeung E, Hu FB, Zhang C (2012). A prospective study of prepregnancy dietary fat intake and risk of gestational diabetes. Am J Clin Nutr.

[27] Djoussé L, Gaziano J, Buring J, Lee I (2009). Egg consumption and risk of type 2 diabetes in men and women. Diabetes Care.

[28] Minooee S, Ramezani Tehrani F, Rahmati M, Mansournia M, Azizi F (2017). Dyslipidemia incidence and the trend of lipid parameters changes in women with history of gestational diabetes: a 15-year follow-up study. Endocrine..

[29] Ryckman KK, Spracklen CN, Smith CJ, Robinson JG, Saftlas AF (2015). Maternal lipid levels during pregnancy and gestational diabetes: a systematic review and meta-analysis. BJOG..

[30] Zhang Y, Lan X, Cai C, Li R, Gao Y, Yang L (2021). Associations between maternal lipid profiles and pregnancy complications: a prospective population-based study. Am J Perinatol.

[31] Khayyatzadeh SS, Kazemi-Bajestani SMR, Bagherniya M, Mehramiz M, Tayefi M, Ebrahimi M (2017). Serum high C reactive protein concentrations are related to the intake of dietary macronutrients and fiber: findings from a large representative Persian population sample. Clin Biochem.

[32] Tall AR, Yvan-Charvet L (2015). Cholesterol, inflammation and innate immunity. Nat Rev Immunol.

[33] Brunham LR, Kruit JK, Pape TD, Timmins JM, Reuwer AQ, Vasanji Z (2007). Beta-cell ABCA1 influences insulin secretion, glucose homeostasis and response to thiazolidinedione treatment. Nat Med.

[34] McGuire S, Scientific Report of the 2015 Dietary Guidelines Advisory Committee (2016). Washington, DC: US Departments of Agriculture and Health and Human Services, 2015. Adv Nutr.

[35] Society CN (2013). Chinese dietary reference intakes.

[36] Su C, Jia X, Wang Z, Wang H, Zhang B (2015). Trends in dietary cholesterol intake among Chinese adults: a longitudinal study from the China Health and Nutrition Survey, 1991-2011. BMJ Open.

[37] Willett W (2013). Nutritional epidemiology.

