# Correction to: Dietary cholesterol and egg intake are associated with the risk of gestational diabetes: a prospective study from Southwest China

**DOI:** 10.1186/s12884-022-04489-2

**Published:** 2022-03-08

**Authors:** Yiqi Zhang, Xi Lan, Fei Li, Hong Sun, Ju Zhang, Run Li, Yan Gao, Hongli Dong, Congjie Cai, Guo Zeng

**Affiliations:** 1 Department of Nutrition and Food Hygiene, West China School of Public Health and West China Fourth Hospital, Sichuan University, Chengdu, 610041 Sichuan China; 2 Department of Clinical Nutrition, Sichuan Provincial Hospital for Women and Children, Chengdu, 610000 Sichuan China; 3 Department of Obstetrics, Sichuan Provincial Hospital for Women and Children, Chengdu, 610000 Sichuan China

**Correction to: BMC Pregnancy Childbirth 22, 45 (2022)**

**https://doi.org/10.1186/s12884-022-04382-y**

After publication of this article [1], it came to our attention that there was an error in Tables 1 and 4. Egg intake in this paper are measured by “number/d”. In Table 1 and part of Table 4, the unit of egg intake was incorrectly wrote as “g/d”. The corrected Tables 1 and 4 are provided below. This error did not impact the conclusions of the article. The authors apologize for this error.

**Table 1:** Characteristics and dietary intake of participants according to quartile of total cholesterol intake

Characteristic	Overall		Quartile of total cholesterol intake(mg/d)								*P* -value
			Q1(≤186.0)		Q2(186.1-332.0)		Q3(332.1-464.0)		Q4(>464.0)		
**N**	1617		408		404		401		404		
Age, years (mean, SD)	28.6	4.1	28.0	3.8	28.5	3.9	29.0	4.4	28.9	4.1	0.001
Prepregnancy BMI, kg/m^2^ (mean, SD)	21.1	3.1	21.2	3.2	21.1	3.1	21.3	3.0	20.9	3.0	0.249
Parity (N, %)											
Primiparous	1157	71.6	290	71.1	286	70.8	293	73.1	288	71.3	0.890
Multiparous	460	28.4	118	28.9	118	29.2	108	26.9	116	28.7	
Family history of diabetes (N, %)	162	10.0	40	9.8	40	9.9	41	10.2	41	10.1	0.997
Education (N, %)											
High school or lower	387	23.9	103	25.2	109	27.0	96	23.9	79	19.6	<0.001
Junior college	590	36.5	175	42.9	135	33.4	146	36.4	134	33.2	
University or higher	640	39.6	130	31.9	160	39.6	159	39.7	191	47.3	
Monthly income of the family per capita, yuan (N, %)											
≤5000	535	33.1	147	36.0	125	30.9	134	33.4	129	31.9	0.215
5000~9999	743	45.9	192	47.1	191	47.3	184	45.9	176	43.6	
≥10000	339	21.0	69	16.9	88	21.8	83	20.7	99	24.5	
Smoking before pregnancy (N, %)	61	3.8	17	4.2	10	2.5	12	3.0	22	5.5	0.119
Alcohol consumption before pregnancy (N, %)	131	8.1	34	8.3	23	5.7	28	7.0	46	11.4	0.021
WGPO, kg (mean, SD)	6.20	3.31	5.64	3.26	6.43	3.25	6.27	3.26	6.44	3.40	0.001
Physical activity, MET^-h^ wk^-1^ (mean, SD)	106.8	46.4	105.3	48.8	108.7	49.0	105.8	41.9	107.4	45.5	0.710
Plasma TG, mmol/L (mean, SD)	1.32	0.50	1.30	0.47	1.31	0.45	1.36	0.52	1.30	0.57	0.257
Plasma TC, mmol/L (mean, SD)	4.14	0.64	4.05	0.66	4.13	0.61	4.17	0.63	4.21	0.67	0.002
Plasma LDL-C, mmol/L (mean, SD)	2.05	0.9	2.03	0.52	2.05	0.48	2.06	0.48	2.04	0.48	0.932
Plasma HDL-C, mmol/L (mean, SD)	1.67	0.34	1.60	0.34	1.66	0.34	1.68	0.32	1.75	0.35	<0.001
GDM (N, %)	571	35.3	118	28.9	146	36.1	151	37.7	156	38.6	0.016
**Dietary intake**											
Eggs, number/d (mean, SD)	0.71	0.58	0.12	0.21	0.49	0.29	0.89	0.31	1.34	0.56	<0.001
Red meats, g/d (mean, SD)	59.5	50.5	39.3	39.0	59.5	41.7	61.0	51.1	78.3	59.7	<0.001
Poultry, g/d (mean, SD)	12.5	29.2	6.3	17.7	11.2	22.4	13.5	29.2	19.3	40.8	<0.001
Fish/shellfish, g/d (mean, SD)	18.6	41.8	6.9	19.3	14.1	33.5	17.6	41.6	35.8	57.8	<0.001
Total dairy products, g/d (mean, SD)	138.5	132.8	93.8	116.2	132.6	129.5	149.4	124.7	178.7	145.0	<0.001
Animal organs, g/d (mean, SD)	5.4	17.3	3.2	14.8	4.6	11.5	4.5	13.6	9.1	25.4	<0.001
Dietary glycaemic load, g/d (mean, SD)	153.3	59.4	147.3	58.7	152.7	53.6	158.4	67.6	154.8	56.7	0.061
Energy intake, kcal/d (mean, SD)	1848.1	530.5	1600.1	486.6	1819.1	510.2	1901.2	475.4	2074.9	535.9	<0.001
Saturated fat, unsaturated fat, g/d (mean, SD)	68.8	24.8	53.0	19.1	66.6	23.5	72.0	20.3	83.6	25.6	<0.001
Animal protein, g/d (mean, SD)	25.4	15.4	12.8	8.2	22.7	10.4	27.3	12.2	39.1	16.4	<0.001
Fibre, g/d (mean, SD)	12.9	6.3	11.9	6.5	12.9	6.7	13.0	5.8	13.7	5.9	<0.001

*BMI* body mass index, *WGPO* Weight gain from pregnancy to OGTT, *TG* triglyceride, *TC* total cholesterol, *HDL-C* high-density lipoprotein cholesterol, *LDL-C* low-density lipoprotein cholesterol, *GDM* gestational diabetes mellitus, *MET* metabolic equivalent

**Table 4:** Associations between maternal dietary egg intake per day and GDM

	Continuous variable (each additional egg/d)		Quartile egg intake (number/d)								*P* for trend
			Q1		Q2		Q3		Q4		
	OR	95%CI	OR	95%CI	OR	95%CI	OR	95%CI	OR	95%CI	
Median, number/d	0.7		0		0.5		1.0		1.3		-
Model 1^a^	1.36	1.14-1.62	1.00	-	1.48	1.11-1.98	1.46	1.09-1.96	1.64	1.22-2.20	0.002
Model 2^b^	1.32	1.11-1.58	1.00	-	1.48	1.11-1.99	1.39	1.03-1.87	1.59	1.18-2.14	0.006
Model 2 plus nutrients^c^											
Saturated fat, unsaturated fat	1.30	1.08-1.56	1.00	-	1.47	1.09-1.97	1.35	1.00-1.84	1.54	1.13-2.09	0.013
Animal protein	1.33	1.10-1.60	1.00	-	1.48	1.11-1.99	1.39	1.02-1.89	1.59	1.16-2.17	0.008
Fibre	1.32	1.11-1.58	1.00	-	1.48	1.11-1.99	1.39	1.03-1.88	1.59	1.18-2.15	0.005
All previous nutrients	1.32	1.10-1.60	1.00	-	1.48	1.10-1.98	1.38	1.02-1.88	1.58	1.16-2.17	0.009
Model 2 plus plasma lipid profiles(mmol/L) ^d^											
TG	1.30	1.10-1.60	1.00	-	1.57	1.16-2.12	1.46	1.07-1.99	1.65	1.20-2.25	0.009
TC	1.31	1.09-1.58	1.00	-	1.61	1.19-2.18	1.45	1.06-1.98	1.64	1.20-2.24	0.011
LDL-C	1.33	1.11-1.60	1.00	-	1.62	1.20-2.19	1.46	1.07-1.99	1.68	1.23-2.30	0.007
HDL-C	1.31	1.09-1.58	1.00	-	1.60	1.19-2.16	1.44	1.06-1.96	1.64	1.20-2.25	0.011
All previous measurements	1.30	1.08-1.56	1.00	-	1.58	1.17-2.13	1.46	1.07-1.99	1.63	1.19-2.23	0.011

*TG* triglyceride, *TC* total cholesterol, *HDL-C* high-density lipoprotein cholesterol, *LDL-C* low-density lipoprotein cholesterol, *GDM* gestational diabetes mellitus

^a^ Crude model

^b^ Adjusted for energy intake, age, prepregnancy BMI, education, income of family, parity, family history of diabetes, smoking before pregnancy, alcohol consumption before pregnancy, physical activity, dietary glycaemic load, weight gain from pregnancy to OGTT

^c^ Nutrients correlated with egg (saturated fat, unsaturated fat, trans fat, animal protein and fibre) were adjusted individually or in combination, in addition to Model 2 covariates

^d^ To determine whether the effect of egg intake on GDM is related to the increase of plasma lipid profiles, the concentrations of plasma TG, TC, LDL-C and HDL-C were further adjusted individually or in combination
