# Supplementary material for: Dietary cholesterol and egg intake are associated with the risk of gestational diabetes: a prospective study from Southwest China
Source: BMC Pregnancy Childbirth. 2022 Jan 17;22:45. doi: 10.1186/s12884-022-04382-y (PMC8764826; doi:10.1186/s12884-022-04382-y)
Supplement: Supplementary file 1 — Additional file 1: Table S1. Characteristics and dietary intake of participants according to quartile of egg intake. Table S2. Associations of total cholesterol and egg intake with blood glucose levels [file 12884_2022_4382_MOESM1_ESM.docx]

**Table S1 Characteristics and dietary intake of participants according to quartile of egg intake**

| Characteristic | Overall | | Quartile of egg intake(number/d) | | | | | | | | *P* -value |
| --- | --- | --- | --- | --- | --- | --- | --- | --- | --- | --- | --- |
|  |  |  | Q1(≤0.24) | | Q2(0.25-0.66) | | Q3(0.67-1.00) | | Q4(>1.00) | |  |
| **N** | 1617 |  | 408 |  | 425 |  | 404 |  | 380 |  |  |
| Age, years (mean, SD) | 28.6 | 4.1 | 28.2 | 4.1 | 28.3 | 3.9 | 29.3 | 4.2 | 28.6 | 4.2 | 0.001 |
| Prepregnancy BMI, kg/m^2^(mean, SD) | 21.1 | 3.1 | 21.0 | 3.2 | 21.3 | 3.0 | 21.2 | 3.1 | 21.1 | 3.1 | 0.535 |
| Parity (N, %) |  |  |  |  |  |  |  |  |  |  |  |
| Primiparous | 1157 | 71.6 | 288 | 70.6 | 303 | 71.3 | 283 | 70.0 | 283 | 74.5 | 0.524 |
| Multiparous | 460 | 28.4 | 120 | 29.4 | 122 | 28.7 | 121 | 30.0 | 97 | 25.5 |  |
| Family history of diabetes (N, %) | 162 | 10.0 | 37 | 9.1 | 46 | 10.8 | 40 | 9.9 | 39 | 10.3 | 0.863 |
| Education (N, %) |  |  |  |  |  |  |  |  |  |  |  |
| High school or lower | 387 | 23.9 | 109 | 26.7 | 109 | 25.6 | 91 | 22.5 | 78 | 20.5 | 0.045 |
| Junior college | 590 | 36.5 | 159 | 39.0 | 158 | 37.2 | 144 | 35.6 | 129 | 33.9 |  |
| University or higher | 640 | 39.6 | 140 | 34.3 | 158 | 37.2 | 169 | 41.8 | 173 | 45.5 |  |
| Monthly income of the family per capita, yuan (N, %) |  |  |  |  |  |  |  |  |  |  |  |
| ≤5000 | 535 | 33.1 | 145 | 35.5 | 136 | 32.0 | 119 | 29.5 | 135 | 35.5 | 0.175 |
| 5000~9999 | 743 | 45.9 | 183 | 44.9 | 210 | 49.4 | 191 | 47.3 | 159 | 41.8 |  |
| ≥10000 | 339 | 21.0 | 80 | 19.6 | 79 | 18.6 | 94 | 23.3 | 86 | 22.6 |  |
| Smoking before pregnancy (N, %) | 61 | 3.8 | 20 | 4.9 | 15 | 3.5 | 7 | 1.7 | 19 | 5.0 | 0.053 |
| Alcohol consumption before pregnancy (N, %) | 131 | 8.1 | 36 | 8.8 | 27 | 6.4 | 35 | 8.7 | 33 | 8.7 | 0.493 |
| WGPO, kg (mean, SD) | 6.20 | 3.31 | 5.98 | 3.23 | 6.12 | 3.38 | 6.43 | 3.34 | 6.26 | 3.27 | 0.241 |
| Physical activity, MET^-h^ wk^-1^(mean, SD) | 106.8 | 46.4 | 108.2 | 49.0 | 107.3 | 46.1 | 104.8 | 47.3 | 106.9 | 42.7 | 0.749 |
| Plasma TG, mmol/L (mean, SD) | 1.32 | 0.50 | 1.29 | 0.44 | 1.35 | 0.50 | 1.29 | 0.49 | 1.33 | 0.58 | 0.248 |
| Plasma TC, mmol/L (mean, SD) | 4.14 | 0.64 | 4.10 | 0.64 | 4.11 | 0.62 | 4.14 | 0.67 | 4.20 | 0.64 | 0.095 |
| Plasma LDL-C, mmol/L (mean, SD) | 2.05 | 0.9 | 2.06 | 0.50 | 2.03 | 0.49 | 2.05 | 0.51 | 2.04 | 0.46 | 0.870 |
| Plasma HDL-C, mmol/L (mean, SD) | 1.67 | 0.34 | 1.62 | 0.35 | 1.64 | 0.33 | 1.68 | 0.35 | 1.74 | 0.34 | <0.001 |
| GDM (N, %) | 571 | 35.3 | 112 | 27.5 | 161 | 37.9 | 148 | 36.6 | 150 | 39.5 | 0.001 |
| **Dietary intake** |  |  |  |  |  |  |  |  |  |  |  |
| Red meats, g/d (mean, SD) | 59.5 | 50.5 | 57.2 | 58.0 | 59.1 | 40.6 | 60.9 | 52.2 | 60.9 | 49.9 | 0.690 |
| Poultry, g/d (mean, SD) | 12.5 | 29.2 | 12.9 | 34.2 | 10.9 | 22.7 | 13.1 | 29.4 | 13.4 | 29.7 | 0.582 |
| Fish/shellfish, g/d (mean, SD) | 18.6 | 41.8 | 14.7 | 37.8 | 17.1 | 33.7 | 19.1 | 43.4 | 23.8 | 51.1 | 0.019 |
| Total dairy products, g/d (mean, SD) | 138.5 | 132.8 | 109.9 | 137.6 | 139.0 | 119.7 | 144.5 | 128.0 | 162.3 | 141.1 | <0.001 |
| Animal organs, g/d (mean, SD) | 5.4 | 17.3 | 5.6 | 22.9 | 5.2 | 11.8 | 5.5 | 18.3 | 5.1 | 14.4 | 0.966 |
| Dietary glycaemic load, g/d (mean, SD) | 153.3 | 59.4 | 154.3 | 64.0 | 152.5 | 52.3 | 150.5 | 57.7 | 155.9 | 63.7 | 0.613 |
| Energy intake, kcal/d (mean, SD) | 1848.1 | 530.5 | 1753.7 | 611.0 | 1815.8 | 471.0 | 1871.0 | 540.7 | 1961.2 | 464.6 | <0.001 |
| Saturated fat, unsaturated fat, g/d (mean, SD) | 68.8 | 24.8 | 61.6 | 28.2 | 66.6 | 20.8 | 71.2 | 25.0 | 76.2 | 22.4 | <0.001 |
| Animal protein, g/d (mean, SD) | 25.4 | 15.4 | 19.0 | 16.1 | 23.1 | 11.0 | 27.9 | 15.9 | 32.3 | 15.1 | <0.001 |
| Total cholesterol, mg/d (mean, SD) | 340.8 | 204.3 | 140.3 | 128.8 | 269.6 | 100.7 | 410.2 | 131.5 | 562.1 | 164.8 | <0.001 |
| Fibre, g/d (mean, SD) | 12.9 | 6.3 | 12.4 | 7.0 | 12.5 | 5.8 | 13.3 | 6.1 | 13.4 | 6.0 | 0.052 |

BMI, body mass index; WGPO, Weight gain from pregnancy to OGTT; TG, triglyceride; TC, total cholesterol; HDL-C, high-density lipoprotein cholesterol; LDL-C, low-density lipoprotein cholesterol; GDM, gestational diabetes mellitus; MET, metabolic equivalent.

**Table S2 Associations of total cholesterol and egg intake with blood glucose levels**

| Variable | FBG, mmol/L | | |  | 1-h PBG, mmol/L | | |  | 2-h PBG, mmol/L | | |
| --- | --- | --- | --- | --- | --- | --- | --- | --- | --- | --- | --- |
|  | *β* | 95% CI | P-value |  | *β* | 95% CI | *P*-value |  | *β* | 95% CI | *P*-value |
| **Total cholesterol intake (mg/d)** |  |  |  |  |  |  |  |  |  |  |  |
| Model 1^a^ | -0.001 | -0.011 to 0.008 | 0.801 |  | 0.082 | 0.038-0.127 | <0.001 |  | 0.071 | 0.033-0.110 | <0.001 |
| Model 2^b^ | -0.002 | -0.013 to 0.009 | 0.753 |  | 0.068 | 0.019-0.117 | 0.007` |  | 0.050 | 0.008-0.092 | 0.020 |
| Model 2 plus nutrients ^c^ |  |  |  |  |  |  |  |  |  |  |  |
| Saturated fat, unsaturated fat | -0.003 | -0.014 to 0.008 | 0.615 |  | 0.055 | 0.004-0.105 | 0.033 |  | 0.050 | 0.001-0.091 | 0.044 |
| Animal protein | -0.004 | -0.017 to 0.009 | 0.521 |  | 0.059 | 0.000-0.117 | 0.049 |  | 0.051 | 0.001-0.101 | 0.047 |
| Fibre | -0.001 | -0.012 to 0.010 | 0.810 |  | 0.064 | 0.015-0.114 | 0.011 |  | 0.048 | 0.006-0.091 | 0.026 |
| All previous nutrients | -0.005 | -0.017 to 0.008 | 0.491 |  | 0.053 | 0.001-0.111 | 0.048 |  | 0.046 | 0.000-0.096 | 0.049 |
| Model 2 plus cholesterol-containing foods ^d^ |  |  |  |  |  |  |  |  |  |  |  |
| Eggs | -0.010 | -0.030 to 0.011 | 0.350 |  | 0.061 | -0.032 to 0.153 | 0.199 |  | 0.040 | -0.040 to 0.119 | 0.327 |
| Red meats | -0.003 | -0.013 to 0.008 | 0.649 |  | 0.066 | 0.017-0.115 | 0.009 |  | 0.050 | 0.008-0.093 | 0.020 |
| poultry | -0.001 | -0.012 to 0.009 | 0.792 |  | 0.068 | 0.018-0.117 | 0.007 |  | 0.052 | 0.009-0.094 | 0.017 |
| Fish/shellfish | -0.002 | -0.013 to0.010 | 0.790 |  | 0.073 | 0.022-0.123 | 0.005 |  | 0.054 | 0.011-0.097 | 0.015 |
| Total dairy products | -0.002 | -0.013 to 0.009 | 0.761 |  | 0.064 | 0.015-0.114 | 0.010 |  | 0.045 | 0.002-0.087 | 0.038 |
| Animal organs | -0.002 | -0.013 to 0.009 | 0.723 |  | 0.068 | 0.019-0.118 | 0.007 |  | 0.049 | 0.007-0.092 | 0.022 |
| **Egg (number/d)** |  |  |  |  |  |  |  |  |  |  |  |
| Model 1^a^ | 0.005 | -0.030 to 0.039 | 0.794 |  | 0.251 | 0.095-0.408 | 0.002 |  | 0.222 | 0.087-0.358 | 0.001 |
| Model 2^b^ | 0.004 | -0.031 to 0.038 | 0.830 |  | 0.191 | 0.034-0.348 | 0.017 |  | 0.147 | 0.012-0.282 | 0.033 |
| Model 2 plus nutrients ^e^ |  |  |  |  |  |  |  |  |  |  |  |
| Total cholesterol | 0.030 | -0.035 to 0.095 | 0.365 |  | 0.027 | -0.268 to 0.322 | 0.858 |  | 0.039 | -0.214 to 0.293 | 0.761 |
| Saturated fat, unsaturated fat | 0.002 | -0.032 to 0.037 | 0.898 |  | 0.168 | 0.010-0.326 | 0.037 |  | 0.127 | 0.007-0.262 | 0.048 |
| Animal protein | 0.002 | -0.034 to 0.038 | 0.911 |  | 0.162 | 0.000-0.324 | 0.049 |  | 0.135 | 0.005-0.374 | 0.042 |
| Fibre | 0.004 | -0.030 to 0.039 | 0.808 |  | 0.186 | 0.029-0.343 | 0.021 |  | 0.144 | 0.009-0.280 | 0.036 |
| All previous nutrients except cholesterol | 0.001 | -0.034 to 0.037 | 0.939 |  | 0.155 | 0.029-0.343 | 0.040 |  | 0.127 | 0.002-0.266 | 0.048 |

FBG, fasting blood glucose; PBG, postprandial blood glucose.

^a^ Crude model

^b^ Adjusted for energy intake, age, prepregnancy BMI, education, income of family, parity, family history of diabetes, smoking before pregnancy, alcohol consumption before pregnancy, physical activity, dietary glycaemic load, weight gain from pregnancy to OGTT

^c^ Nutrients correlated with dietary cholesterol (saturated fat, unsaturated fat, trans fat, animal protein and fibre) were adjusted individually or in combination, in addition to Model 2 covariates.

^d^ To determine if certain cholesterol-containing foods were major determinants for the associations, eggs, red meat, poultry, fish and shellfish, total dairy products and edible offal were adjusted individually.

^e^ Nutrients correlated with egg (dietary cholesterol, saturated fat, unsaturated fat, trans fat, animal protein and fibre) were adjusted individually or in combination, in addition to Model 2 covariates.
